# More Is Actually Less: Practitioners' Perspective of Unnecessary Medical Testing in Saudi Arabian Emergency Departments

**DOI:** 10.7759/cureus.62384

**Published:** 2024-06-14

**Authors:** Atheer F AlSulami, Mohammed A AlGhamdi, Amro M Gaafar, Anas F Hamam

**Affiliations:** 1 Emergency Medicine, King Fahad Armed Forces Hospital, Jeddah, SAU; 2 Emergency Medicine, King Abdullah Medical Complex, Jeddah, SAU

**Keywords:** emergency department testing, overutilization, unnecessary ancillary testing, unnecessary imaging, over-testing

## Abstract

Background

The overuse of medical testing, be it ancillary testing or imaging, has been identified as a problem in all healthcare systems in the world. As the Kingdom of Saudi Arabia marches towards the 2030 vision of healthcare transformation, we have sought to get a perspective on medically unnecessary tests being conducted in Saudi Arabian emergency departments (EDs), the reasons behind this phenomenon, and possible solutions to it.

Methods

This is a cross-sectional survey among emergency medicine physicians (EMPs) working in Saudi Arabian EDs, taken through a self-filled online questionnaire, about their ordering habits, what they believe to be unnecessary testing in their practice, the practice of their colleagues, and other Saudi EMPs as a whole. Subjects have also been asked about the reasons why such practices are occurring and possible solutions to reduce such overuse of unnecessary tests in Saudi EDs.

Results

A total of 182 EMPs were surveyed from the different regions of the Kingdom, and CT head for patients presenting with asymptomatic stroke, and asymptomatic TBI were the most overused scans (both 44%). The most overused advanced body imaging was CT kidney-ureters-bladder (KUB) at 41.5%, while the most overused ancillary tests were complete blood count (CBC) and liver transaminases. The most common reason for the practice was found to be fear of medicolegal proceedings (70.9%). Continuous education of EMPs and increasing ED staffing were found to be the most helpful solutions to reduce unnecessary testing in the ED (70.9% and 67%, respectively).

Conclusion

It is clear from our data that overuse of medical tests and imaging is still a prominent practice. CT head in asymptomatic patients seems to be the most commonly overused imaging in Saudi EDs. Ancillary testing and unnecessary ordering of CBCs and transaminases seem to stem from fear of EMPs from legal consequences. More control over medical test ordering needs to be exercised to reduce these practices.

## Introduction

In recent years, the overuse of medical testing and imaging has become somewhat of an emerging trend worldwide [1]. The use of unnecessary ancillary testing, and imaging that do not contribute anything to patient outcomes and, in some cases, may lead to patient harm, is considered a major factor in driving the cost of healthcare up [2,3]. Emergency departments (EDs) in particular are susceptible to issues of this nature because, unlike other healthcare venues, they deal with people who are in acute conditions and pain, with high expectations, minimal to non-existing documentation, and expecting resolution of their clinical conditions, in a very brief time frame [4,5].

The “Choosing Wisely” campaign was launched worldwide in 2012 with the expressed purpose of reducing investigations, imaging, and procedures that were not medically indicated. Previous evidence showed that, while most physicians are aware of the campaign, the majority did not explicitly know of its recommendations and thus did not fully implement it [6]. The “Choosing Wisely” campaign was adopted in the Kingdom of Saudi Arabia by the Saudi Patient Safety Center in 2019 [7].

In accordance with the 2030 vision directive of the Kingdom of Saudi Arabia, the healthcare landscape has been in a state of flux. Emergency medical care and services are at the core of this transformation. As the demand for high-quality specialized emergency care increases, the volume of diagnostic testing and imaging has also been going up [8-10]. This increasing trend of unnecessary testing is caused by multiple factors. Fear of medicolegal litigation is one of the major factors that cause physicians to order medically unnecessary tests.

Known as “Defensive Medicine”, this is the attitude adopted by some physicians in an effort to protect themselves from unjustified and frequent patient complaints, which unfortunately some healthcare facilities over-escalate. They go on to blame the practitioner for not ordering tests, which are actually not indicated, only to appease the patient or their insurance carrier [4,11]. Other factors that contribute to the observed increase in unnecessary testing include the desire of the patient, and their subsequent satisfaction rating, or lack thereof. Wanting to expedite outpatient (OPD) procedures, fear of missing a diagnosis that is rare and remote, and the misguided thought that more testing is synonymous with comprehensive care all contribute to driving the cost of healthcare up [10,11]. It is for this reason that we have decided to conduct a survey to get a perspective on what the personnel working in the EDs in the Kingdom of Saudi Arabia think about the ordering of unnecessary tests and imaging that is prevalent in their departments, its causes, and possible solutions to its reduction.

## Materials and methods

This was a cross-sectional study that was conducted in 2024 through a self-administered online survey that was distributed by email, through 12 emergency departments in the Kingdom of Saudi Arabia that have emergency medicine residency training programs.

Sample size calculation and sampling

For a confidence level of 90%, an expected population of 500 physicians, and a margin of error of 0.05, the required sample size as calculated by an online calculator (https://www.qualtrics.com/blog/calculating-sample-size/) was 176 subjects.

Validation

Our survey was taken and adapted from Kanzaria et al. 2015. The questionnaire in that study was validated in a process outlined in their publication [4].

Primary objectives

The primary objectives are to assess emergency medicine physicians' (EMPs) perception of the volume of over-testing in head and body CT scans, as well as laboratory blood work; to investigate the causes that most likely cause the EMPs to order unnecessary investigations; and to investigate the ways that would most likely be effective in reducing unnecessary investigations.

Secondary objectives

The secondary objectives are to perform subgroup analysis with respect to the EMPs' gender, training level, and regions of practice and to look for differences in responses with respect to these parameters.

Inclusion criteria

The inclusion criteria are residents enrolled in the Emergency Medicine Residency Training Program and board-certified and non-board-certified EMPs working in EDs.

Exclusion criteria

The exclusion criteria are any non-physicians working in EDs and all other non-emergency medical staff.

Data analysis

The data collected were analyzed using the Statistical Product and Service Solutions (SPSS, v.26; IBM SPSS Statistics for Windows, Armonk, NY). Proportions were noted, and the Mann-Whitney Z-test and Kruskal-Wallis H-test were used to measure the qualitative data. Due to the non-normal distribution of data, the non-parametric Pearson correlation test was applied for all subgroup analyses to ascertain the statistical significance of the findings in the subgroup analysis. With regards to the quantitative data (the assessment of the subject on unnecessary testing they were ordering, the Student t-test was implemented.

## Results

Demographics

The survey was answered by 189 subjects, and seven subjects declined to participate. Data from 182 emergency physicians was used in the analysis of this study. Our sample was 62% male, and 84% of the subjects were under the age of 35 years. Additionally, 24.7% of the sample was made up of board-certified EMPs, while the junior and senior emergency program residents comprised 41% and 30% of the sample, respectively. The rest were general physicians working in the ED, with no specialization certification. Our sample was mainly taken from physicians working in the western region of the Kingdom, while the central, western, and eastern provinces contributed 63%, 23%, and 11%, respectively (Table 1).

**Table 1:** Demographic data PGY = Program Year; BC-EMP = Board Certified Emergency Physician; GP = General Physician; PEM = Pediatric Emergency Physician

Topic	Parameter	N (%)
Gender	Male	113 (62.1)
	Female	69 (37.9)
Age	< 30 years	116 (63.0)
	31-35 years	37 (20.2)
	36-40 years	11 (6.0)
	41-45 years	11 (6.0)
	46-50 years	7 (3.8)
	> 50 years	0 (0)
Region	Central Region	42 (23.1)
	Western Region	115 (63.2)
	Eastern Province	20 (11.0)
	Northern Province	1 (0.5)
	Southern Region	4 (2.2)
Years of practice	< 3 years	83 (45.6)
	3-5 years	59 (32.4)
	6-10 years	15 (8.2)
	> 10 years	25 (13.7)
Training level	PGY 1	46 (25.3)
	PGY 2	30 (16.5)
	PGY 3	22 (12.1)
	PGY 4	32 (17.6)
	BC-EMP	45 (24.7)
	GP	6 (3.3)
	PEM	1 (0.5)

Overuse in head imagining

**Figure 1:**
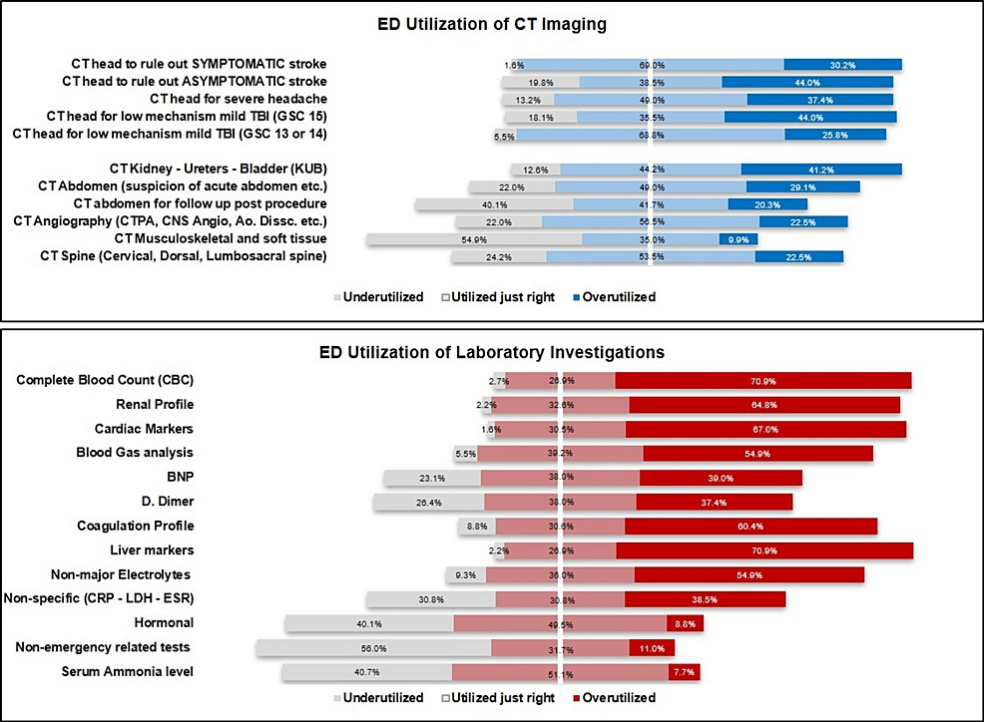
Utilization of advanced imaging and ancillary laboratory and point-of-care (POC) testing in the ED

We asked our subjects about the utilization of advanced imaging (i.e., CT scans) of the head in various scenarios. Additionally, 30.2% reported that CT head in the setting of symptomatic stroke was being overused. If the patient did not have clear symptoms of stroke (i.e., had only a tingling sensation but no motor weakness, etc.), 44% of EMPs thought that CT head was being ordered unnecessarily. Only 38.5% reported that CT was utilized in an appropriate manner. Thus, the presence of symptoms that were in keeping with neurological stroke reduced unnecessary head imaging by about 14%. In patients presenting with headaches, half of EMPs thought that the use of CT head was being used appropriately 49%, while 37.4% thought that the imaging was being overused. With regards to mild traumatic brain injury (TBI), in patients presenting with a GCS of 15/15, 44% of EMPs thought that CT head was being overused, while 18.1% thought it was underused. In similar patients where the GCS was 13-14 (still in the definition of mild TBI), the presence of a drop in the GCS justified 18.2% of the scans, and only 25.8% of EMPs thought that the scan was being overused (Figure 1).

Overuse in body imagining

According to our data, the most overused imaging was CT kidney-ureter-bladder (CT KUB) at 41%. Half of all EMPs thought that CT abdomen with contrast, to rule out surgical emergencies, was being utilized properly, while 30% thought it was being overused, and the rest thought it was being underutilized. In patients who have had laparotomy for one reason or another, and presented to the ED, post-procedure with abdominal pain, 40.1% of EMPs thought that CT abdomen was being ordered for them inappropriately (Figure 1).

With regards to CT angiography (mainly CT pulmonary angiography, CT CNS angiography, and CT Aortic angiography), 22% thought these studies were overused, and 22% thought they were underused, while the rest thought they were being utilized just right. Finally, musculoskeletal CT (i.e., knee, limbs, etc.) was thought to be underused by 54.9% of EMPs, while CT spine showed similar numbers to those of CT angiography (Table 2).

**Table 2:** Investigation utilization in the ED CT = Computed Tomography; TBI = Traumatic Brain Injury; GCS = Glasgow Coma Scale; KUB = Kidney-Ureter-Bladder; AoD = Aortic Dissection; CTPA = Computed Tomography Pulmonary Angiography; CNS = Central Nervous System; BUN = Blood Urea Nitrogen; Tn_c_I = Cardiac Troponin I; Tn_c_T = Cardiac Troponin T; CKMB = Creatinine Kinase Muscle-Brain; PT = Prothrombin Time; PTT = Partial Thromboplastin Time; INR = International Normalized Ratio; AST = Aspartate Aminotransferase; ALT = Alanine Aminotransferase; GGT = Gamma-Glutamyl Transferase; ALP = Alkaline Phosphatase; CRP = C-Reactive Protein; LDH = Lactate Dehydrogenase; ESR = Erythrocyte Sedimentation Rate; T_3_ = Triiodothyronine; T_4_ = Thyroxine; TSH = Thyroid Stimulating Hormone; TIBC = Total Iron binding Capacity; Vit D = Calciferol

Parameter	Way overutilized	Overutilized	Utilized properly	Underutilized	Way underutilized	Mean	SD	Utility
	N (%)	N (%)	N (%)	N (%)	N (%)			
CT head to rule out stroke (in the presence of symptoms)	16 (8.8)	39 (21.4)	124 (68.1)	1 (0.5)	2 (1.1)	3.363	0.698	Overutilized
CT head to rule out stroke (in the ABSENCE of symptoms)	11 (6.0)	69 (37.9)	66 (36.3)	27 (14.8)	9 (4.9)	3.253	0.953	Underutilized
CT head for severe headache	6 (3.3)	62 (34.1)	90 (49.5)	22 (12.1)	2 (1.1)	3.264	0.756	Underutilized
CT head for low mechanism mild TBI (GSC 15)	14 (7.7)	66 (36.3)	69 (37.9)	25 (13.7)	8 (4.4)	3.291	0.951	Overutilized
CT head for low mechanism mild TBI (GSC 13 or 14)	9 (4.9)	38 (20.9)	125 (68.7)	8 (4.4)	2 (1.1)	3.242	0.662	Underutilized
Parameter	Way overutilized	Overutilized	Utilized properly	Underutilized	Way underutilized	Mean	SD	Utility
	N (%)	N (%)	N (%)	N (%)	N (%)			
CT Kidney - Ureters - Bladder (KUB).	19 (10.4)	56 (30.8)	84 (46.2)	21 (11.5)	2 (1.1)	3.379	0.063	Underutilized
CT Abdomen (suspicion of acute abdomen, ischemia, etc.)	6 (3.3)	47 (25.8)	89 (48.9)	34 (18.7)	6 (3.3)	3.071	0.062	Underutilized
CT abdomen for follow-up (post-procedure, mass/tumor, etc.)	4 (2.2)	33 (18.1)	72 (39.6)	46 (25.3)	27 (14.8)	2.675	0.074	Underutilized
CT Angiography (AoD, CTPA, CNS Angio, Ischemic limb, etc.)	8 (4.4)	33 (18.1)	101 (55.5)	36 (19.8)	4 (2.2)	3.027	0.059	Underutilized
CT Musculoskeletal and soft tissue (Hip, knee, wrist, ankle)	2 (1.1)	16 (8.8)	64 (35.2)	68 (37.4)	32 (17.6)	2.384	0.067	Underutilized
CT Spine (C-spine, Dorsal spine, Lumbosacral spine)	7 (3.8)	34 (18.7)	97 (53.3)	41 (22.5)	3 (1.6)	3.005	0.059	Underutilized
Parameter	Way overutilized	Overutilized	Utilized properly	Underutilized	Way underutilized	Mean	SD	Utility
	N (%)	N (%)	N (%)	N (%)	N (%)			
Complete Blood Count (CBC)	73 (40.1)	56 (30.8)	48 (26.4)	3 (1.6)	2 (1.1)	4.071	0.911	Overutilized
Renal Profile (serum creatinine, BUN)	50 (27.5)	68 (37.4)	60 (33.0)	2 (1.1)	2 (1.1)	3.890	0.860	Overutilized
Cardiac Markers (Tn_c_I, Tn_c_T, CKMB)	35 (19.2)	87 (47.8)	57 (31.3)	2 (1.1)	1 (0.5)	3.841	0.759	Overutilized
Blood Gas analysis	47 (25.8)	53 (29.1)	72 (39.6)	9 (4.9)	1 (0.5)	3.747	0.918	Overutilized
B-type natriuretic peptide	26 (14.3)	45 (24.7)	69 (37.9)	29 (15.9)	13 (7.1)	3.231	1.103	Underutilized
D. Dimer	21 (11.5)	47 (25.8)	66 (36.3)	32 (17.6)	16 (8.8)	3.137	1.111	Underutilized
Coagulation Profile (PT, PTT, INR)	43 (23.6)	67 (36.8)	56 (30.8)	14 (7.7)	2 (1.1.)	3.742	0.943	Overutilized
Liver markers (AST, ALT, GGT, ALP, Bilirubin, Albumin)	41 (22.5)	88 (48.4)	49 (26.9)	4 (2.2)	0 (0.0)	3.912	0.760	Overutilized
Non-major Electrolytes (PO_4_, Mg)	32 (17.6)	68 (37.4)	65 (35.7)	15 (8.2)	2 (1.1)	3.621	0.907	Overutilized
Non-specific (CRP - LDH - ESR)	30 (16.5)	40 (22.0)	56 (30.8)	43 (23.6)	13 (7.1)	3.170	1.175	Underutilized
Hormonal (T_3_, T_4_, TSH)	3 (1.6)	13 (7.1)	93 (51.1)	53 (29.1)	20 (11.0)	2.593	0.841	Underutilized
Non-emergency related tests (TIBC, Ferritin, Vit. D)	7 (3.8)	13 (7.1)	60 (33.0)	34 (18.7)	68 (37.4)	2.214	1.139	Underutilized
Serum Ammonia level	3 (1.6)	11 (6.0)	94 (51.6)	38 (20.9)	36 (19.8)	2.489	0.933	Underutilized

Overuse in laboratory and point-of-care (POC) tests

When asked about an array of ED blood tests, the top five tests that most EMPs (i.e., more than 60% of subjects agreed on) being overused and inappropriately ordered were (1) complete blood counts (CBC) 70.9%; (2) liver transaminases 70.9%; (3) cardiac markers 67%; (4) renal profile 64.8%; and (5) coagulation profile 60.4% (Figure 1). According to half the subjects surveyed, blood gas analysis and non-major electrolytes (i.e., phosphate, magnesium, etc.) were being inappropriately ordered at 54.9% (Table 2).

What Percentage of Unnecessary Testing Do You Think You and Your Colleagues Order?

**Figure 2:**
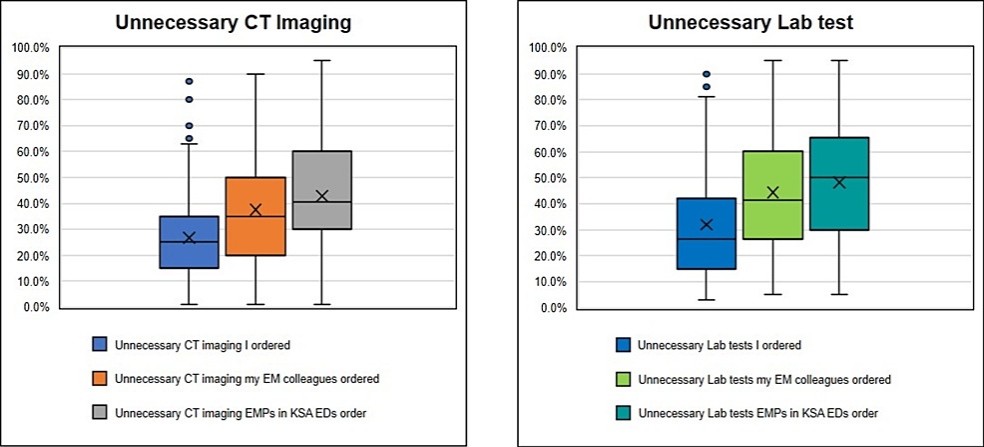
Self-reported unnecessary imaging and testing in EDs

We asked our subjects to estimate the percentage (out of 100) of unnecessary imaging and laboratory testing that they thought they were ordering, that they thought that their colleagues, who work with them in the same department were ordering, and that EMPs in KSA EDs were ordering in general. Although such a question is entirely subjective, it does however show the perspective of the personnel working in the KSA EDs on this matter. It is worth noting that EMPs said that they thought that 37.5% of CTs and 44.3% of laboratory tests ordered by their colleagues were medically unnecessary. However, when they were asked to rate their own ordering, they reported that 26.8% of CT scans and 32% of laboratory tests were unnecessary on average (Figure 2, Table 3). Worthy of note also is that female EMPs reported that they order more unnecessary images and blood work than their male counterparts by at least 5% (p-value <0.001).

**Table 3:** EMPs' perspective of the percentages of unnecessary investigations that they, their colleagues, and other Saudi EMPs order in the KSA EDs

	Mean			Standard Deviation			1^st^ Quartile			Median			3^rd^ Quartile			IQR		
Gender	Male	Female	Both	Male	Female	Both	Male	Female	Both	Male	Female	Both	Male	Female	Both	Male	Female	Both
Unnecessary CT imaging on average I order (%)	26.19	27.74	26.77	16.98	17.67	17.21	14.5	15	15	25	29	25	34.5	40	35	20	25	20
Unnecessary CT imaging my colleagues order (%)	37.57	37.38	37.5	20.64	22.65	21.36	20.5	20	20	35	36	35	50	52.5	50	29.5	32.5	30
Unnecessary CT imaging EMPs in KSA EDs order (%)	44.08	40.59	42.84	19.25	22.66	20.49	30	20	30	40	41	40.5	60	60	60	30	40	30
Unnecessary Lab tests on average I order (%)	29.99	35.42	32.05	19.48	24.87	21.78	15.5	14	15	25	33	26.5	37.5	52.5	42.25	22	38.5	27.25
Unnecessary Lab tests my colleagues order (%)	43.72	45.28	44.31	22.16	24.67	23.09	26	27.5	26.5	41	42	41.5	60	65	60.25	34	37.5	33.75
Unnecessary Lab tests EMPs in KSA EDs order (%)	47.99	48.43	48.16	22.17	23.87	22.77	30	30.5	30	48	50	50	62	69.5	65.5	32	39	35.5
p-value	< 0.001			< 0.001			N/A			< 0.001			N/A			< 0.001		

What, in Your Opinion, Are the Reasons for Ordering Unnecessary Investigations in the ED?

**Figure 3:**
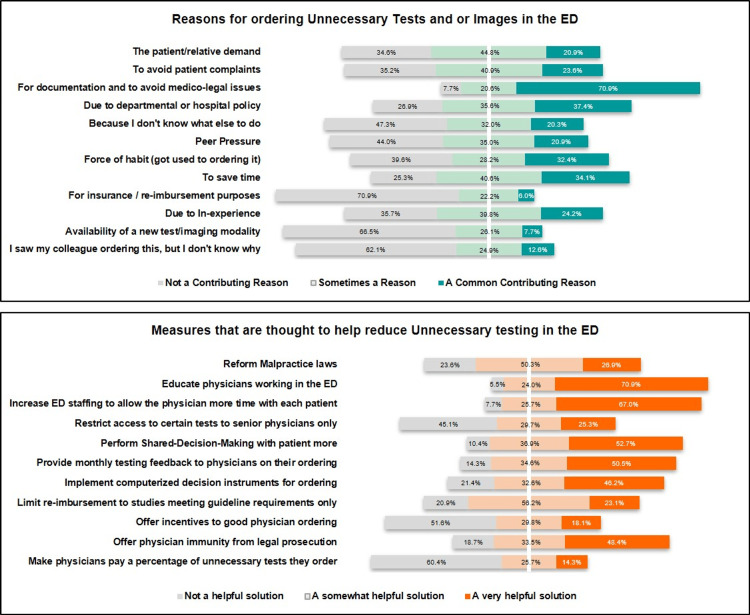
Reasons and possible solutions for unnecessary testing in EDs

Our subjects were asked about the reasons they thought that unnecessary testing was happening in the Saudi Arabian EDs and what possible solutions they thought were helpful in reducing or even resolving the problem. The most commonly chosen reasons for unnecessary testing were documentation to avoid medico-legal issues and conformation to departmental and institutional clinical pathways (70.9% and 37.4%, respectively). Some of the unlikely reasons were the reimbursement of insurance claims, the availability of a new testing modality, and emulating other colleagues (70.9%, 66.5%, and 62.1%, respectively; Table 4, Figure 3).

**Table 4:** Unnecessary ED testing possible reasons and solutions

Parameter	Almost always a reason	Often a reason	Sometimes a reason	Rarely a reason	Almost never a reason	Mean	SD	Contribution
	N (%)	N (%)	N (%)	N (%)	N (%)			
The demand of the patient or the relative	6 (3.3)	32 (17.6)	81 (44.5)	44 (24.2)	19 (10.4)	2.791	0.964	Contributing Reason
To avoid patient complaints	10 (5.5)	33 (18.1)	75 (41.2)	44 (24.2)	20 (11.0)	2.830	1.029	Contributing Reason
For documentation and to avoid medico-legal issues	62 (34.1)	67 (36.8)	39 (21.4)	10 (5.5)	4 (2.2)	3.951	0.988	Contributing Reason
Due to departmental and/or hospital policy	22 (12.1)	46 (25.3)	65 (35.7)	36 (19.8)	13 (7.1)	3.154	1.097	Contributing Reason
Because I don't know what else to do	4 (2.2)	33 (18.1)	59 (32.4)	56 (30.8)	30 (16.5)	2.588	1.036	Non-contributing
Peer Pressure from department colleagues	4 (2.2)	34 (18.7)	64 (35.2)	51 (28.0)	29 (15.9)	2.632	1.031	Non-contributing
Force of habit (got used to ordering it)	16 (8.8)	43 (23.6)	51 (28.0)	41 (22.5)	31 (17.0)	2.846	1.216	Contributing Reason
To save the patient time	19 (10.4)	43 (23.6)	74 (40.7)	20 (11.0)	26 (14.3)	3.049	1.158	Contributing Reason
For insurance/reimbursement purposes	1 (0.5)	10 (5.5)	42 (23.1)	48 (26.4)	81 (44.5)	1.912	0.971	Non-contributing
Due to Inexperience or lack of knowledge	6 (3.3)	38 (20.9)	73 40.1)	34 (18.7)	31 (17.0)	2.747	1.073	Contributing Reason
Availability of a new test or imaging modality	0 (0.0)	14 (7.7)	47 (25.8)	55 (30.2)	66 (36.3)	2.049	0.965	Non-contributing
I saw my colleague ordering this, but I don't know why	7 (3.8)	16 (8.8)	46 (25.3)	55 (30.2)	58 (31.9)	2.225	1.107	Non-contributing
Parameter	Extremely Helpful	Very Helpful	Somewhat helpful	Not Helpful	Not Helpful at All	Mean	SD	Utility
	N (%)	N (%)	N (%)	N (%)	N (%)			
Reform Malpractice laws	18 (9.9)	31 (17.0)	90 (49.5)	30 (16.5)	13 (7.1)	3.060	1.009	Not Helpful
Educate physicians working in the emergency departments	59 (32.4)	70 (38.5)	43 (23.6)	10 (5.5)	0 (0.0)	3.978	0.885	A good Solution
Increase ED staffing to allow the physician more time with patients	59 (32.4)	63 (34.6)	46 (25.3)	11 (6.0)	3 (1.6)	3.901	0.981	A good Solution
Restrict access to certain tests to senior physicians only	8 (4.4)	38 (20.9)	54 (29.7)	56 (30.8)	26 (14.3)	2.703	1.087	Not Helpful
Perform Shared-Decision-Making with patients more	23 (12.6)	73 (40.1)	67 (36.8)	15 (8.2)	4 (2.2)	3.527	0.896	A good Solution
Provide monthly feedback to physicians on their ordering	32 (17.6)	60 (33.0)	64 (35.2)	16 (8.8)	10 (5.5)	3.484	1.055	A good Solution
Implement computerized decision instruments for ordering	21 (11.5)	63 (34.6)	59 (32.4)	25 (13.7)	14 (7.7)	3.286	1.085	A good Solution
Limit reimbursement to studies meeting guideline requirements	4 (2.2)	38 (20.9)	102 (56.0)	30 (16.5)	8 (4.4)	3.000	0.801	Not Helpful
Offer incentives to good physician ordering	5 (2.7)	28 (15.4)	55 (30.2)	53 (29.1)	41 (22.5)	2.467	1.086	Not Helpful
Offer physician immunity from legal prosecution	35 (19.2)	53 (29.1)	60 (33.0)	27 (14.8)	7 (3,8)	3.451	1.080	A good Solution
Make physicians pay a percentage of unnecessary tests they order	4 (2.2)	22 (12.1)	46 (25.3)	34 (18.7)	76 (41.8)	2.143	1.157	Not Helpful

Finally, when asked about possible solutions to reduce such practices, six solutions were proposed as helpful: (1) education of EMPs (70.9%); (2) increasing the ED staff to allow more physician-patient time (67%); (3) increasing shared decision making (SDM) with patients (52.7%); (4) providing the EMPs with monthly feedback on their ordering (50.5%); (5) providing EMPs with immunity from prosecution (48.4%); and (6) implementing computerized ordering algorithms (46.2%). All numerical data can be found in Table 4.

Subgroup analyses

Subgroup analyses were carried out in our study with a previous *apriori* to look into differences with respect to gender, training level, and region of practice of the subjects surveyed. The only item that showed differences in opinion, based on the gender of the EMP surveyed, was the ordering of the renal profile. Additionally, 76.9% of female EMPs reported the overuse of this test, while only 57.5% of male EMPs said it was being ordered unnecessarily (p-value = 0.006). The training levels of the subjects in our study did not seem to reveal any differences in responses worthy of note, in any item. However, the main differences were in the region of practice of the subjects surveyed.

The use of head CT in patients presenting with mild TBI, and a GCS of 13-14, was reported by central, western, and eastern region subjects, to be overordered unnecessarily by 33.3%, 24.6%, and 9.5% of EMPs, respectively (*p-*value = 0.015). B-type natriuretic peptide (BNP) was noted by central and western region EMPs to be overused by 41-45% of subjects, while only 20% of EMPs practicing in the eastern region noted this test’s overuse (p-value = 0.007). Finally, with regards to helpful solutions to reduce unnecessary testing, increasing the ED staffing was voted as a very helpful solution to this problem by 76.2% of central region EMPs, 67% of western region EMPs, and only 40% of eastern region EMPs (p-value = 0.006).

## Discussion

It is clear from our data that overuse and unnecessary testing are still going on in Saudi Arabian EDs at significant rates. The items that EMPs report to be overused most in head imaging are the CT in patients presenting with asymptomatic stroke and those presenting with mild asymptomatic TBI (GCS 15/15). When compared with the same scans in symptomatic patients, understandably, the presence of symptoms goes a long way to legitimizing testing where the rate of unnecessary testing goes down by 14% for stroke and 18.2% for mild TBI patients.

Regarding body imaging, CT KUB is the most reported unnecessary overused scan (41.2%). This is about 11% more than any other body imaging scan, which is consistent with previously reported evidence by Ghoshal et al. and Kasi et al. [12,13]. For ancillary testing, complete blood count and liver transaminases were reported most to be overused and medically unnecessary. This is also in keeping with previous international evidence [14,15].

As expected, when subjects were asked about their own ordering habits and whether or not the test that they were asking for was medically necessary, all EMPs enrolled in our study reported their own overuse of testing at least 10-12% less than they estimated their colleagues or other EMPs in the Kingdom were ordering. Reasons for unnecessary testing according to our data were mainly due to fear of medicolegal complaints. This is in full agreement with what was published by Alalshiekh et al. in 2022 [16]. Solutions to reduce unnecessary medical testing, according to our data, also agreed with the solutions proposed in the “Choosing Wisely” campaign, the three main items being (1) physician continuous education; (2) increasing the ED staffing, as to allow more time for physicians to spend with each patient; and (3) implementing more shared-decision-making [7,16].

Subgroup analyses on the whole did not show any major or statistically significant differences in responses when compared with regards to gender or level of training. However, there were some differences when comparisons were made with respect to the region of practice in the Kingdom. However, due to the low representation of the eastern region relative to the central or western regions, we cannot be certain that such findings can be confidently taken as true, even though statistical significance was established. If such differences are true, the differences seen in the overuse of CT head in TBI could be due to differences in frequencies of such cases seen in the two regions (such data were not checked as it is outside the scope of this study). The relative frequency of such cases can be a confounding factor that may explain the data seen. This also may be the cause of why EMPs in the central region voted on increasing the staffing in the ED as the most helpful solution to reduce the problem of unnecessary testing 76.2%, while only 40% of EMPs in the eastern region noted this solution to be helpful. The differences in ED patient influx in both regions have not been compared or taken into account.

Limitations

The following are the limitations of the study:

(i) This study is of a cross-sectional nature, and as such, it represents a snapshot at the time the study was run of the opinions of the physicians who took part in it. In time, such opinions can change, and departments may have a change in the physicians who are staffing them, leading to different conclusions from those stated by our data.

(ii) The physicians practicing in the eastern region represented only half of those who were practicing in the central region, while those in the western region were three times the number of those in the central region. Hence, the eastern region could have been under-represented in this study.

## Conclusions

From our data, we conclude that the most overused imaging studies according to EMPs were CT head in asymptomatic strokes, and asymptomatic mild TBIs, as well as CT KUB. Complete blood counts and liver transaminases were still the most overused ancillary tests ordered in the ED. The most likely cause of unnecessary testing was fear of medicolegal complaints and the implementation of institutional policy. The most helpful solutions to reduce such unnecessary testing in most EMPs' opinions were to educate physicians further, increase the ED staffing, and increase SDM with patients.

## References

[1] Shaffer VA, Scherer LD (2018). Too much medicine: behavioral science insights on overutilization, overdiagnosis, and overtreatment in health care. PIBBS.

[2] Korenstein D, Falk R, Howell EA, Bishop T, Keyhani S (2012). Overuse of health care services in the United States: an understudied problem. Arch Intern Med.

[3] Romano MJ, Segal JB, Pollack CE (2015). The association between continuity of care and the overuse of medical procedures. JAMA Intern Med.

[4] Kanzaria HK, Hoffman JR, Probst MA, Caloyeras JP, Berry SH, Brook RH (2015). Emergency physician perceptions of medically unnecessary advanced diagnostic imaging. Acad Emerg Med.

[5] Newton EH (2017). Addressing overuse in emergency medicine: evidence of a role for greater patient engagement. Clin Exp Emerg Med.

[6] Maughan BC, Baren JM, Shea JA, Merchant RM (2015). Choosing wisely in emergency medicine: a national survey of emergency medicine academic chairs and division chiefs. Acad Emerg Med.

[7] (2024). Choosing wisely. https://choosingwisely.spsc.gov.sa/docs/choosing_wisely_one_pager_en.png.

[8] Albejaidi FM (2010). Healthcare system in Saudi Arabia: an analysis of structure, total quality management and future challenges. JAPSS.

[9] Rahman R, Qattan A (2021). Vision 2030 and sustainable development: state capacity to revitalize the healthcare system in Saudi Arabia. Inquiry.

[10] Khattab E, Sabbagh A, Aljerian N, Binsalleeh H, Almulhim M, Alqahtani A, Alsalamah M (2019). Emergency medicine in Saudi Arabia: a century of progress and a bright vision for the future. Int J Emerg Med.

[11] Cadamuro J, Gaksch M, Wiedemann H (2018). Are laboratory tests always needed? Frequency and causes of laboratory overuse in a hospital setting. Clin Biochem.

[12] Ghoshal N, Gaikstas G (2021). CT KUB scans for renal colic: optimisation of scan range to reduce patient radiation burden. Radiography (Lond).

[13] Kasi A, Steffens T, Starkey D, Braithwaite V (2021). The proportion of computed tomography kidneys, ureters and bladder (CTKUB) scans that comply with scan extent protocol in an emergency department: a clinical audit and dose ramification study. J Med Radiat Sci.

[14] Calvin Young, Francesca Brundisini, Sarah C. McGill (2024). Identifying overused lab tests in hospital settings: a Delphi study. https://www.cadth.ca/identifying-overused-lab-tests-hospital-settings-delphi-study.

[15] Bhardwaj A (2019). Excessive ancillary testing by healthcare providers: reasons and proposed solutions. J Hospital Med Management.

[16] Alalshaikh A, Alyahya B, Almohawes M (2022). Emergency medicine physicians’ views on providing unnecessary management in the emergency department. Open Access Emerg Med.

